# Fully-Coupled FSI Computational Analyses in the Ascending Thoracic Aorta Using Patient-Specific Conditions and Anisotropic Material Properties

**DOI:** 10.3389/fphys.2021.732561

**Published:** 2021-10-20

**Authors:** Emanuele Vignali, Emanuele Gasparotti, Simona Celi, Stéphane Avril

**Affiliations:** ^1^BioCardioLab, UOC Bioingegneria, Fondazione Toscana Gabriele Monasterio, Massa, Italy; ^2^Mines Saint-Etienne, Université de Lyon, INSERM, SaInBioSE U1059, Saint-Étienne, France

**Keywords:** fluid structure interactions (FSI), fully-coupled FSI, hemodynamics, ascending thoracic aortic aneurysms, small on large

## Abstract

Computational hemodynamics has become increasingly important within the context of precision medicine, providing major insight in cardiovascular pathologies. However, finding appropriate compromise between speed and accuracy remains challenging in computational hemodynamics for an extensive use in decision making. For example, in the ascending thoracic aorta, interactions between the blood and the aortic wall must be taken into account for the sake of accuracy, but these fluid structure interactions (FSI) induce significant computational costs, especially when the tissue exhibits a hyperelastic and anisotropic response. The objective of the current study is to use the Small On Large (SOL) theory to linearize the anisotropic hyperelastic behavior in order to propose a reduced-order model for FSI simulations of the aorta. The SOL method is tested for fully-coupled FSI simulations in a patient-specific aortic geometry presenting an Ascending Thoracic Aortic Aneurysm (aTAA). The same model is also simulated with a fully-coupled FSI with non-linear material behavior, without SOL linearization. Eventually, the results and computational times with and without the SOL are compared. The SOL approach is demonstrated to provide a significant reduction of computational costs for FSI analysis in the aTAA, and the results in terms of stress state distribution are comparable. The method is implemented in ANSYS and will be further evaluated for clinical applications.

## 1. Introduction

Computer fluid dynamics and structural analysis of the cardiovascular system have become fundamental for evaluating the effects of severe diseases (Lee et al., 2014; Gray and Pathmanathan, 2018). Numerical methods are increasingly well adopted for the calculation of blood flow distributions, intramural stresses and several other biomarkers. Particular attention has always been paid to large vessels such as the ascending thoracic aorta (Martin et al., 2015; Capellini et al., 2018). Hemodynamics simulations can be used to augment the information from medical imaging and to improve the knowledge about different pathologies. For example, ascending thoracic aortic aneurysms (aTAA) are a critical cardiovascular pathology where a local bulge is formed in the ascending thoracic aortic segment. The main complication associated with this pathology is the risk of dissection, which can be very sudden and lead to patient sudden death. Current clinical practice is based on the surveillance of aTAA reaching a critical diameter, as the risk of dissection averagely increases with aneurysm size. However, on a patient-specific basis, many factors can increase or decrease the risk of dissection. Hemodynamics and the interactions between the blood and the aortic wall are ones of these causes and computational approaches aim at making patient-specific predictions of these factors and of their role in aTAA rupture (Capellini et al., 2020; De Nisco et al., 2020). The current trend is personalized medicine, where patient-specific parameters are introduced at different levels including morphology, fluid dynamics conditions and tissue mechanics (Condemi et al., 2017; Boccadifuoco et al., 2018a; Fanni et al., 2020).

In the current state of the art, Computational Fluid Dynamics (CFD) (Capellini et al., 2018) and Fluid-Structure Interactions (FSI) (Bianchi et al., 2017; Campobasso et al., 2018) simulations have already been widely used for aTAA analysis. Both approaches were proven to be effective for predicting complex vascular flows during aTAA pathology progression. FSIs are particularly important, as CFD neglects the mechanical alterations of the wall that are at the root of aTAA rupture (Iliopoulos et al., 2009). Nevertheless, given the high level of complexity, assumptions usually need to be introduced. The FSI approach can be simplified by assuming a single one-way coupling for the aTAA, in which the fluid domain transmits the force without receiving any displacement from the structural domain (Lantz et al., 2011). However, in the current state of the art, two-way or fully-coupled FSI methods stand out as the most reliable approach to evaluate aTAA progression. Several studies in which aTAA material properties were assumed as linear elastic were reported (Boccadifuoco et al., 2018b), while others adopt hyperelastic isotropic or anisotropic models (Mendez et al., 2018; Bäumler et al., 2020). The linear approximation is justified by the relatively small deformations occurring between the diastolic and systolic phase of a cardiac cycle. Linearization provides an effective way to significantly reduce the computational cost of the simulation. It is well accepted that aTAA are not only hyperelastic but also strongly anisotropic (Martufi et al., 2014). The state of the art reports fiber-based constitutive models (Vignali et al., 2020), which are the gold standard. Nevertheless, these models remain very onerous for computational FSI simulations. Therefore, there is a pressing need for a trade-off between model approximation and complexity. A suitable fully-coupled FSI approach for aTAA analysis should introduce an acceptable level of approximation without neglecting the intrinsic anisotropy and local variations in the aortic tissue. Beyond anisotropy and hyperelasticity of the aTAA, another aspect to be taken into account is the presence of pre-stress in the vessel. A number of studies dedicated to aTAA assumed the diastolic phase as unloaded (Pons et al., 2020), although it is reasonable to consider stresses induced by the diastolic pressure (Moireau et al., 2012; Bäumler et al., 2020).

To include these effects of anisotropy, hyperelasticity and pre-stress, the Small on large (SOL) linearization appears as appropriate. The basic principle of SOL is to superimpose small deformations on deformed models, obtained from large displacements. Large displacements and strains occur between the unloaded zero pressure and the diastolic phase only, while the diastole-systole cycles exhibit small deformations. By assuming small displacements and strains, point-wise linearization, depending on the stress state at diastole, is imposed by also accounting for anisotropy (Baek et al., 2007).

Our objective in the current study is to implement the SOL framework for FSI analyses of real aTAA cases. A first section is dedicated to introducing the theory. Then, the SOL framework is described. Patient-specific morphology and mechanical data are used to set up a fully-coupled FSI simulation including the SOL approach. The framework encompasses linearization of local material properties, inclusion of pre-stress and anisotropy effects. The same case is then simulated with a fully-coupled FSI without SOL linearization. The results in terms of stress maps and computational times are then presented and compared. Eventually, the performances of the proposed SOL approach are discussed for future clinical applications.

## 2. Theory

The SOL theory was previously introduced in a small number of publications (Baek et al., 2007; Figueroa et al., 2009; Ramachandra and Humphrey, 2019). In the following, we briefly describe the basic principles of this approach, along with the background of continuum mechanics and constitutive modeling.

**Figure 1:**
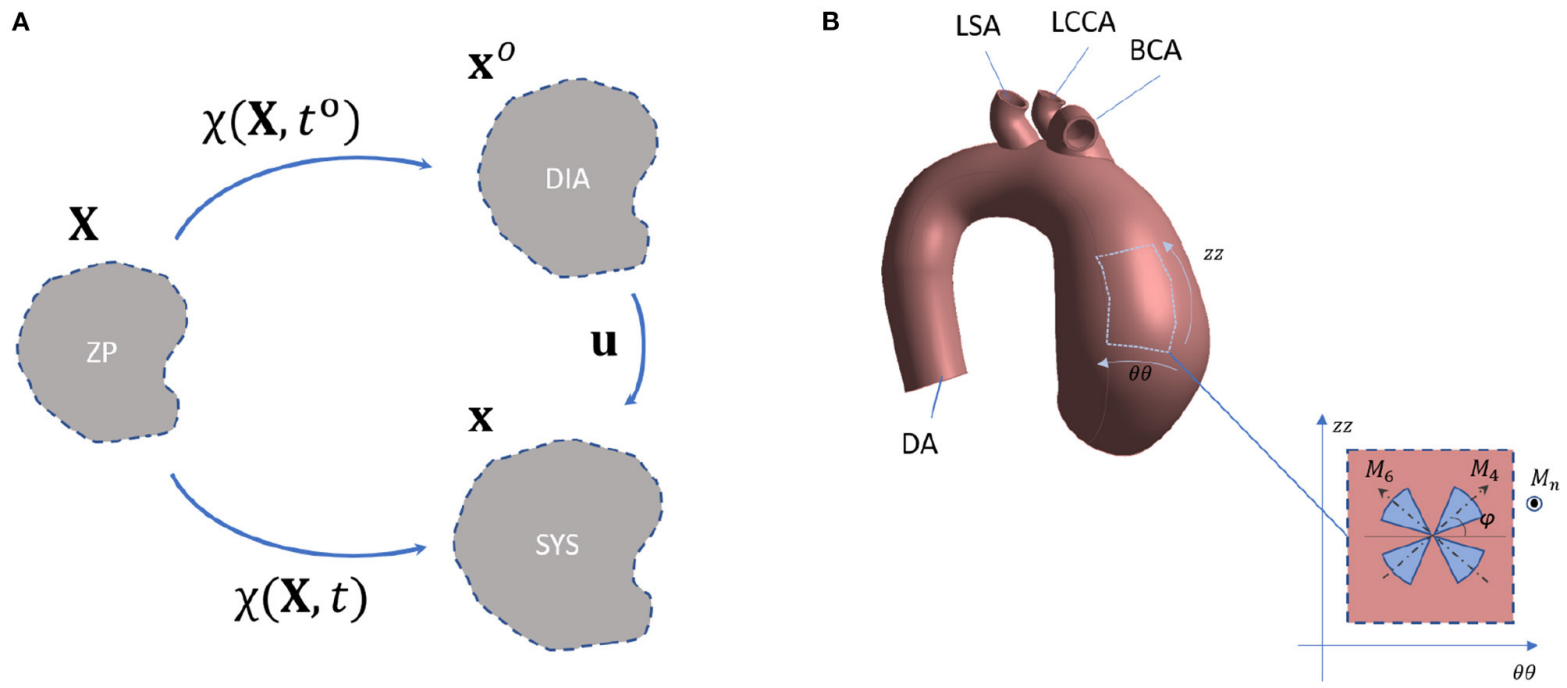
**(A)** Schematic of the mechanical configurations according to SOL theory and **(B)** representation of the main directions of fibers in the aTAA specimen according to the fiber-based constitutive model.

Let us consider a vessel in an initial undeformed configuration **X** transformed according to a mapping function at a given time *t*, χ(**X**, *t*). An additional intermediate state is introduced, according to the mapping function χ(**X**, *t*^*o*^) (Figure 1A). The intermediate state is assumed to be stressed. For the specific case of aTAA simulation, the initial, intermediate and final configurations are assumed as the zero-pressure (ZP), diastolic (DIA) and systolic (SYS) configurations, respectively. The main assumption of the SOL approach is the hypothesis of large deformations between ZP and DIA and hypothesis of small deformations between DIA and SYS. The position vectors are related to the mapping functions according to


(1) $$\begin{array}{l} \text{x}=\chi \left(\text{X},t\right),\;{\text{x}}^{o}=\chi \left(\text{X},t^{o}\right), \end{array}$$

where **x** and **x**^*o*^ are the position vectors in the final and intermediate state, respectively. From the mapping functions, the deformation gradients can be derived such as


(2) $$\begin{array}{l} \text{F}=\frac{\delta \text{x}}{\delta \text{X}}=\frac{\delta \chi \left(\text{X},t\right)}{\delta \text{X}},\;{\text{F}}^{o}=\frac{\delta {\text{x}}^{o}}{\delta \text{X}}=\frac{\delta \chi \left(\text{X},t^{o}\right)}{\delta \text{X}}. \end{array}$$

The deformation gradient occurring between the DIA and the SYS configurations can be defined as


(3) $$\begin{array}{l} {\text{F}}^{*}=\frac{\delta \text{x}}{\delta {\text{x}}^{o}}=\text{I}+\frac{\delta \text{u}}{\delta {\text{x}}^{o}}=\text{I}+\text{H}, \end{array}$$

where **u** is the displacement vector between the DIA and the SYS configurations and **H** is the corresponding displacement gradient. **H** can be expressed as the sum of two main components:


(4) $$\begin{array}{l} \text{H}=\epsilon +\Omega \end{array}$$

where **ϵ** and **Ω** are the small strain and rotation occurring during the deformation and represent, respectively, the symmetric and skew-symmetric part of **H**, according to:


(5) $$\begin{array}{l} \epsilon =\frac{1}{2}\left(\text{H}+{\text{H}}^{T}\right),\;\Omega =\frac{1}{2}\left(\text{H}-{\text{H}}^{T}\right) \end{array}$$

The Cauchy stress tensor **σ** is now introduced according to


(6) $$\begin{array}{l} \sigma =\hat{\sigma }-p\text{I},\;\hat{\sigma }=\text{F}\text{S}{\text{F}}^{T},\;\text{S}=2\frac{\delta W\left(\text{C}\right)}{\delta \text{C}}, \end{array}$$

where *p* is a Lagrangian multiplier accounting for vessel incompressibility, **S** is the second Piola-Kirchhoff stress tensor, *W* is the strain energy density function of the tissue and **C** = **F**^*T*^**F** is the right Cauchy-Green deformation tensor. Both the deformation and stress tensor in the SYS configuration can be expressed as


(7) $$\begin{array}{l} \sigma =\sigma^{*}+\sigma^{o},\;\text{F}={\text{F}}^{*}{\text{F}}^{o}, \end{array}$$

where σ^*^ is the stress change occurring between the DIA and SYS configurations, while **σ**^*o*^ is the stress tensor in the DIA configuration (pre-stress). It can be expressed, according to Equation (6), as **σ**^*o*^ = (**F**^*o*^**S**^*o*^**F**^*o*^*T*) − *p*^*o*^**I**. By considering (Equations 3, 6, and 7), it is possible to define


(8) $$\begin{array}{l} \sigma =\left({\text{F}}^{o}+\text{H}{\text{F}}^{o}\right)\left({\text{S}}^{*}+{\text{S}}^{o}\right)\left({\text{F}}^{o}T+{\text{F}}^{o}T{\text{H}}^{T}\right)-\left(p^{*}+p^{o}\right)\text{I}, \end{array}$$

where *p* was split into an intermediate (*p*^*o*^) and final (p^*^) part and S^*^ is the change of the second Piola-Kirchhoff stress tensor, which can be expressed according to the theory of elasticity as:


(9) $$\begin{array}{l} {\text{S}}^{*}=\left(\frac{\delta \text{S}}{\delta \text{C}}{|}_{{\text{C}}^{o}}\right){\text{C}}^{*},\;{\text{C}}^{*}=2{\text{F}}^{oT}\epsilon {\text{F}}^{o} \end{array}$$

The higher order terms of **H** resulting from Equation (8) are neglected under the assumption of small deformations. The final approximate expression for the Cauchy stress tensor is given by:


(10) $$\begin{array}{l} \sigma ≃\sigma^{o}-p^{*}\text{I}+\text{H}\hat{\sigma }+\hat{\sigma }{\text{H}}^{T}+{\text{F}}^{o}{\text{S}}^{*}{\text{F}}^{oT} \end{array}$$

By considering (Equations 6, 9, and 10) can be written in terms of tensor components as:


(11) $$\begin{array}{l} \sigma_{ij}=\sigma_{ij}^{o}-p^{*}\delta_{ij}+H_{ik}{\hat{\sigma }}^{o}o_{kj}+{\hat{\sigma }}_{ik}^{o}H_{jk}\\ +\left(4F_{iA}^{o}F_{jB}^{o}F_{kP}^{o}F_{lQ}^{o}\frac{\delta^{2}W}{\delta C_{AB}\delta C_{PQ}}{|}_{{\text{C}}^{o}}\right)\epsilon_{kl} \end{array}$$

The last term of Equation (11) was obtained by evaluating the second derivative of the strain energy density function with respect to the right Cauchy-Green deformation tensor. The derivative is evaluated at the deformation state defined by the tensor **C**^*o*^ = **F**^*oT*^**F**^*o*^, defined between the zero-pressure to the DIA configuration. In addition, by considering the two components of the tensor **H**, fourth-order tensors can be introduced in Equation (11):


(12) $$\begin{array}{l} \sigma_{ij}=\sigma_{ij}^{o}-p^{*}\delta_{ij}+{\mathbb{C}}_{ijkl}^{o}o\epsilon_{kl}+{𝔻}_{ijkl}^{o}\Omega_{kl} \end{array}$$

where ℂ^*o*^ is a fourth-order linearized stiffness tensor, obtained from the derivative of the second Piola-Kirchhoff tensor and accounting for small elastic strains, which can be expressed in its matrix form as


(13) $$\begin{array}{l} {\mathbb{C}}_{ijkl}^{o}=\delta_{ik}\sigma_{lj}^{o}+\sigma_{jk}^{o}\delta_{il}+4F_{iA}^{o}F_{jB}^{o}F_{kP}^{o}F_{lQ}^{o}{\frac{\delta^{2}W}{\delta C_{AB}\delta C_{PQ}}|}_{{\text{C}}^{o}} \end{array}$$

the fourth-order tensor 𝔻^*o*^, accounting for small rotations, is instead given by:


(14) $$\begin{array}{l} {𝔻}_{ijkl}^{o}=\delta_{ik}\sigma_{lj}^{o}+\sigma_{jk}^{o}\delta_{il} \end{array}$$

It is worth noting that the first two factors of ℂ^*o*^, as expressed in Equation (13), account for the presence of pre-stress **σ**^*o*^, while the third factor is the linearized stiffness component, deriving directly from the strain energy density function of the material. Linearization is performed for evaluating the derivative at the deformation tensor **C**^*o*^, defined between the zero-pressure to the DIA configuration. Equation (12) expresses the stress tensor in the SYS configuration as a combination of pre-stress, derived from the DIA configuration, and linearized response of the tissue, according to the linearized stiffness tensor expressed in Equation (13).

At this point, to completely define the SOL method, it is sufficient to determine a suitable strain energy density function *W* to be linearized, according to Equation (13). To this purpose, a two-fiber-family hyperelastic and anisotropic model with fiber dispersion is introduced (Niestrawska et al., 2019). The strain energy density function expression is written such as


(15) $$\begin{array}{l} W=\frac{c}{2}\left({\text{I}}_{1}-3\right)+\frac{k_{1}}{2k_{2}}\underset{i=4,6}{\sum }\left[e^{\left(k_{2}^{i}{\left({\text{I}}_{i}^{*}-1\right)}^{2}\right)}-1\right], \end{array}$$

where *c*, *k*_1_, and *k*_2_ are the model parameters representing the isotropic matrix stiffness, the fiber family stiffness and the fiber stiffening factor, respectively. **I**_**1**_ is the first invariant of **C** and ${\text{I}}_{i}^{*}$ is the pseudo-invariant defined according to the generalized structure tensor **G**_***i***_, such as


(16) $$\begin{array}{l} {\text{G}}_{i}=A\text{I}+B{\text{M}}_{i}⊗{\text{M}}_{i}+\left(1-3A-B\right){\text{M}}_{op}⊗{\text{M}}_{op}. \end{array}$$

The generalized structure tensor is expressed as a function of the out-of-plane direction vector, **M**_***op***_, and the vectors defining the *i*-th family preferential direction, **M**_***i***_. The two fiber families in the vessel tissue are assumed to be symmetric and to lay in the plane defined by the circumferential (θθ) and longitudinal (*zz*) direction (Figure 1B). According to this hypothesis, the **M**_***i***_ vectors can be completely defined by the fiber family angle parameter α_*i*_, according to


(17) $$\begin{array}{l} {\text{M}}_{i}=\cos\alpha_{i}{\text{e}}_{1}+\sin\alpha_{i}{\text{e}}_{2}, \end{array}$$

where **e**_**1**_ and **e**_**2**_ are the vectors of the circumferential and longitudinal direction, respectively. The fiber dispersion is modeled in Equation (16) with the parameters *A* and *B*. The expression for *A* and *B* depends on the parameters *k*_*ip*_ and *k*_*op*_, representing the in-plane and out-of-plane dispersion factors, according to

(18) $$\begin{array}{l} A=2k_{ip}k_{op},\;B=2k_{op}\left(1-2k_{ip}\right). \end{array}$$

The reported constitutive model was implemented in the SOL framework to compute the pre-stress component from the ZP-DIA large deformation step, while it was linearized for the DIA-SYS small deformation step.

## 3. Materials and Methods

### 3.1. Geometry and Material Properties

An aTAA patient-specific morphology was reconstructed based on CT data. The data were acquired *in vivo* on a patient harboring an aTAA, just before surgical intervention at the Heart Hospital of Massa. The CT images were obtained with a 320-detector scanner (Toshiba Aquilon One, Toshiba, Japan) by adopting a iodinated contrast medium. The clinical data in the diastolic phase were analyzed through a threshold segmentation algorithm to obtain the aortic morphology including the aTAA section, the supra-aortic branches and the descending aorta.

After assessing the morphology, material properties were defined on the basis of *ex vivo* tissue characterization. In particular, a tissue sample was collected from the aTAA after the surgical intervention. A square specimen of about 30 × 30 mm was cut in the outer curvature region of the harvested aTAA tissue. The specimen was maintained at room temperature in physiological saline solution and tested 3 h after tissue harvesting. Freezing processes were avoided in order to maintain the microstructure integrity. Biaxial tests were carried out with the OptiMech2 setup (Vignali et al., 2021). The obtained stress-stretch data from the tests were fitted according to the constitutive model of Equation (15) in order to be implemented in the simulation framework.

### 3.2. Reverse Displacement Algorithm

**Figure 2:**
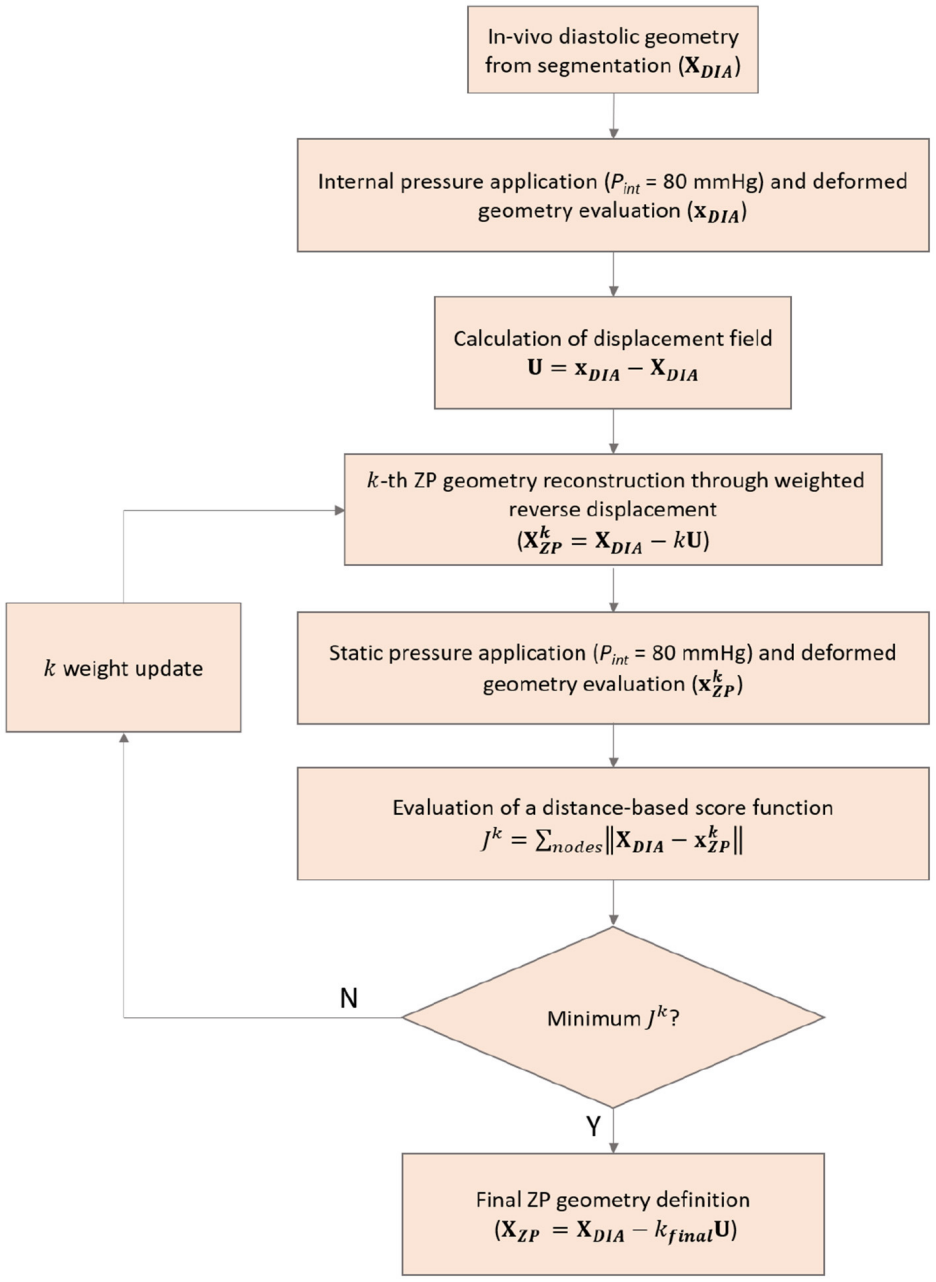
Schematic of the reverse displacement algorithm used for the ZP geometry estimation.

Before performing FSI simulations, the unloaded ZP configuration was firstly reconstructed. To obtain the ZP configuration from the *in vivo* diastolic configuration, a reverse-displacement method was implemented, as introduced by Raghavan et al. (2006). The material properties were the ones fitted against the biaxial test data. The software ANSYS mechanical was used for all the computations. Fiber-based constitutive relationships according to Equation (15) were implemented in the ANSYS environment with a custom made FORTRAN script. The reverse displacement method workflow is represented schematically in Figure 2. The main principle is to find a scalar quantity to describe the size change of the aTAA structure on the basis of a distance score, evaluated with respect to the DIA configuration. The complete description of the method is provided in Raghavan et al. (2006). Briefly, the main steps are the following:

- The diastolic pressure (*P*_*int*_ = 80 mmHg) was applied to the diastolic spatial configuration (**X**_*DIA*_). The model was assumed hyperelastic, according to Equation (15). The corresponding deformed configuration was obtained (**x**_*DIA*_).
- The resulting displacement field was calculated from the difference between the deformed and reference configuration (**U** = **x**_*DIA*_ − **X**_*DIA*_).
- The displacement field was reversed and weighted with a scalar parameter *k* (range 0.5–2) to model the size change. The resulting displacement field was applied to the reference configuration to obtain the *k*-th ZP configuration candidate (${\text{X}}_{ZP}^{k}={\text{X}}_{DIA}-k\text{U}$).
- The *k*-th ZP geometry candidate was then pressurized with a static simulation by applying an internal pressure corresponding to the diastolic condition (*P*_*int*_ = 80 mmHg). The material model was assumed hyperelastic, according to Equation (15). The deformed configuration corresponding to *k*-th ZP geometry candidate was obtained (${\text{x}}_{ZP}^{k}$).
- A score function *J*^*k*^ was calculated for the *k*-th candidate configuration. The score was defined as:
  (19) $$\begin{array}{l} J^{k}=\overset{N}{\sum }\|{\text{X}}_{DIA}-{\text{x}}_{ZP}^{k}\| \end{array}$$
  where *N* is the number of mesh nodes and the operator ||.|| represents the Euclidean distance.
- Previous steps 3, 4 and 5 were repeated by modifying the *k* parameter until a minimum of the *J*^*k*^ is reached, with *k* = *k*_*final*_. At this point, the final candidate for the ZP configuration is defined as ${\text{X}}_{ZP}^{final}={\text{X}}_{DIA}-k_{final}\text{U}$

The configuration minimizing the score from the algorithm was chosen as the ZP configuration for the next simulation steps. The comparison between the initial candidate and the final pressurized ZP configuration was calculated in terms of distance from the DIA configuration.

### 3.3. SOL-Linearized Simulation

**Figure 3:**
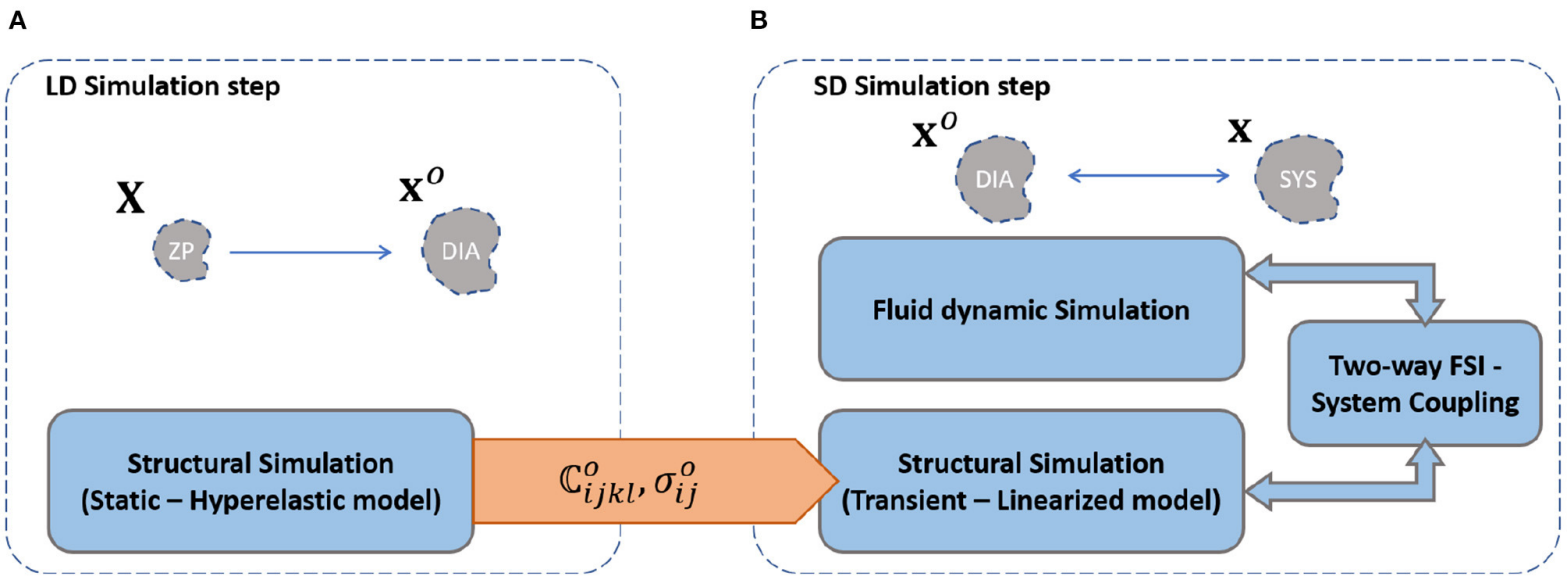
Schematic of the workflow for the main simulation steps of SOL-linearized Simulation: **(A)** Large Deformation and **(B)** Small Deformation step.

After assessing the ZP configuration, the SOL-Linearized simulation was setup. All the steps for the SOL linearization procedure are summarized in Figure 3. Software ANSYS mechanical and ANSYS Fluent were used for all the simulations. All computation were achieved with the same machine (Processor : Intel Core i9-9900, 4 cores, CPU@3.10 GHz; RAM: 32 GB). The main steps are given by the Large Deformation and the Small Deformation steps.

#### 3.3.1. Large Deformation Step

The Large Deformation (LD) step was set up first (Figure 3A). The deformation occurring between the ZP and the DIA configuration was assumed as large. For this reason, the linearization hypothesis cannot be made and the patient-specific hyperelastic material model was implemented for the LD simulation step. The load was modeled as static with a pressure of 80 mmHg, representing the physiological diastolic load. The pressure was applied on the internal wall of the ZP aortic geometry. Regarding boundary conditions, the descending aorta was fixed at the end. At the ends of each supra aortic vessel and at the plane of the aortic valve, we manually defined a cylindrical reference system. For each of these boundaries, the longitudinal and circumferential displacements were fixed, while the radial displacement was left free, as implemented in previous studies (Celi and Berti, 2014; Campobasso et al., 2018). The stress and strain tensors evaluated from the LD simulation step were processed in order to obtain the pre-stress and the linearized tangent stiffness tensors for each mesh element, according to Equation (13). Results were processed with a custom Matlab script. Stiffness outliers, expected in the neighborhood of constrained nodes, were ignored for the successive simulation step.

#### 3.3.2. Small Deformation Step

The results from the LD step were adopted for the implementation of the Small Deformation simulation step (Figure 3B). Unlike the previous phase, since the deformation occurring from the diastolic to the systolic condition was small, the linearization was allowed. Briefly, the DIA aortic geometry was loaded with a fully-coupled FSI simulation to obtain the corresponding systolic configuration. The fluid and structural domains were discretized with a mesh of about 850,000 tetrahedral elements. The FSI coupling was implemented through the System Coupling component, which controls the simulation execution in ANSYS. The component uses an Arbitrary Lagrangian Eulerian method (Degroote et al., 2010). The method implements a bidirectional transfer at the domain interface: the fluid pressure is transmitted to the solid domain and the incremental solid displacement to the fluid domain. The System Coupling was used to configure the fluid and structural simulations execution. In particular, each time step was subdivided in up to a maximum of 30 coupling iterations. At each coupling iteration, the exchange of loads and displacement occurs up to convergence. Mechanical load exchange was managed with both relaxation function and ramping, to improve the stability of the simulation (Chimakurthi et al., 2018). The convergence criterion was set according to a maximum root-mean-square residual of 0.01 for both fluid and solid simulation. The time step value for the simulation was set equal to 0.001 s (Campobasso et al., 2018).

Concerning the fluid dynamics simulation section, the blood was assumed as a Newtonian fluid with a density of 1060 kg/m^3^ and a viscosity of 0.0035 Pa s. A physiological velocity profile was imposed as inlet at the aortic valve level. In particular, the profile was obtained from 4D flow MRI datasets, performed by means of a 3T MR-scanner (Ingenia, Philips Medical Systems, Amsterdam, Netherlands). Given the aortic valve section, the imposed velocity resulted in a cardiac output of 4.8 l/min with an heart rate of 77 bpm. A total of 5 cardiac cycles were simulated. The pressure conditions were maintained at the brachio-cephalic artery (BCA), the left common coronary artery (LCCA), the left subclavian artery (LSA) and descending aorta (DA) outlets according to the three-element Windkessel model. The values of the lumped parameters were calculated according to a state-of-the-art algorithm (Boccadifuoco et al., 2018a) and set to maintain a pressure range of 0–40 mmHg. The resulting proximal resistance (*R*_*p*_), capacitance (*C*) and distal resistance (*R*_*d*_) parameters are reported in Table 1. As pre-stress was already present, the fluid dynamic load at diastole was assumed to be zero. A dynamic meshing condition was imposed at the fluid domain wall to permit the exchange of data with the structural domain without mesh degradation.

**Table 1:** Table reporting the parameters for the Windkessel models for the SOL FSI simulation.

	***R*_*p*_ (Kg/m^**4**^s)**	***C* (Kg/m^**4**^s^**2**^)**	***R*_***d***_ (Kg/m^**4**^s)**
BCA	5.1e7	1.5e-9	8.1e8
LCCA	8.1e7	9.7e-10	1.3e9
LSA	4.7e7	1.7e-9	7.4e8
DA	1.3e7	6.1e-9	2.0e8

Concerning structural simulations, the tissue was modeled according to the linearized model from the previous LD step. For each mesh element, the stiffness tensor and the pre-stress were introduced to account for both anisotropy and diastolic pre-load, respectively. In particular, an anisotropic linear material model was set for the whole domain, while the material properties were automatically re-defined for each element before proceeding with the actual FSI simulation. The linearized stiffness tensors were evaluated with a specific Matlab script by processing the previous large deformation step results. Then, the same script generates an APDL routine to re-assign the material properties of each element. Finally, the APDL routine is loaded within the ANSYS environment. The load condition was imposed on the basis of the data exchange of the FSI. The same boundary condition constraints from the previous LD simulation were imposed for this step.

The pressure and flow conditions were calculated and post-processed. The circumferential and longitudinal stress distributions were evaluated along a cardiac cycle and presented as a result. The computation times were recorded to permit a comparison with the non-linear simulation.

### 3.4. Non-linear Simulation

To compare the performances and results of the SOL-linearized simulation with the standard approach, the same aortic geometry was simulated without assuming small deformations occurring between diastole and systole and without imposing the linearization of the material model. In this case, the fiber-based constitutive model with patient-specific parameters was still assumed. To allow comparison, the non-linear simulation was computed with the same machine from the previous step, and the domains were discretized with approximately the same number of tetrahedral elements. Briefly, the ZP aortic geometry was loaded with a fully-coupled FSI simulation to obtain the corresponding systolic configuration. Similarly to the SD step of the SOL simulation, the FSI simulation execution was managed with the System Coupling component of ANSYS. The same time step value, maximum number of coupling iterations and under relaxation function were adopted. As pre-stress was absent and the simulation was managed in a single step for this case, the fluid dynamics load conditions were adapted to consider the diastolic load. In particular, the Windkessel model parameters were calculated according to a state-of-art algorithm, already used for the SD step of the SOL procedure, in order to maintain a physiological pressure range of 80 - 120 mmHg, at regime. The resulting *R*_*p*_, *C* and *R*_*d*_ parameters for BCA, LCCA, LSA and DA branches are reported in Table 2. The difference between the lumped parameters from the SOL FSI and the non-linear FSI was expected, as the pressure range imposed in the SOL FSI was lower than in the non-linear simulation. Conversely, the non-linear FSI requires the full physiological range of 80–120 mmHg, as the starting point is the unloaded configuration without pre-stress. For the inlet condition at the aortic valve, the flow profile was imposed by adding an initial constant flow followed by a total of 5 cardiac cycles (the same as in SOL FSI simulation). The initial constant flow was added to permit gradual pressure increase. Concerning the structural part, the same boundary conditions as the SOL-Linearized simulation steps were imposed.

**Table 2:** Table reporting the parameters for the Windkessel models for the non-linear FSI simulation.

	***R*_*p*_ (Kg/m^**4**^s)**	***C* (Kg/m^**4**^s^**2**^)**	***R*_*d*_ (Kg/m^**4**^s)**
BCA	6.4e7	1.1e-9	9.5e8
LCCA	9.5e7	3.7e-10	1.5e9
LSA	3.7e7	2.1e-9	8.5e8
DA	1.9e7	6.0e-9	4.0e8

The circumferential and longitudinal stress distributions were calculated along a cardiac cycle and compared with the distributions from the previous SOL simulations.

## 4. Results

**Figure 4:**
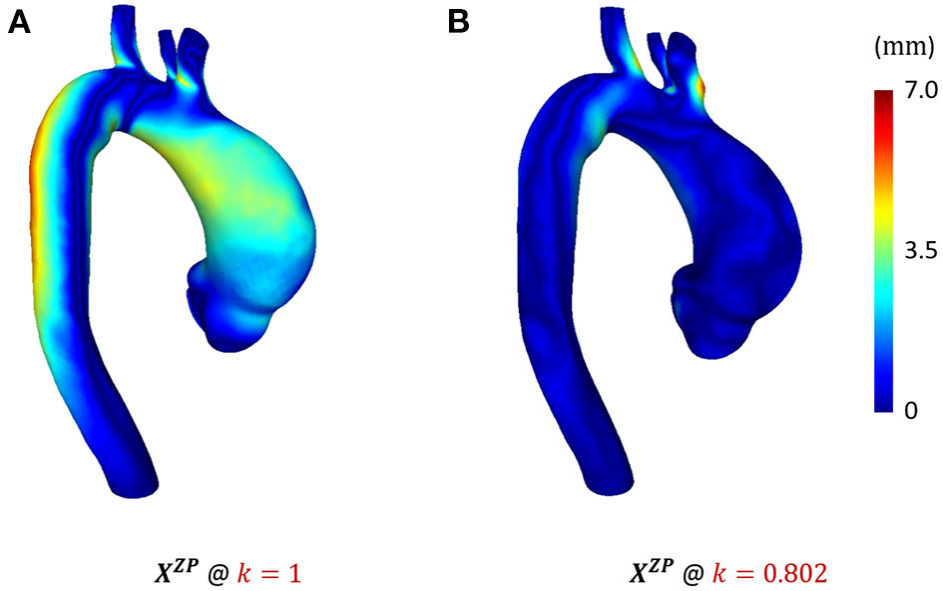
Results in terms of distance from diastolic target geometry after pressurization of ZP configuration candidates: **(A)** *k* = 1 (initial guess) and **(B)** *k* = 0.802.

In Figure 4A, we show the ZP configuration obtained with our reverse displacement algorithm using a scale factor of 0.802. The ZP configuration is significantly different of the *in vivo* diastolic configuration shown in Figure 4B. The average distance between the two configurations in the aTAA region was eventually 0.2 mm, whereas it was only of 1.6 mm with the initial guess of ZP configuration.

**Figure 5:**
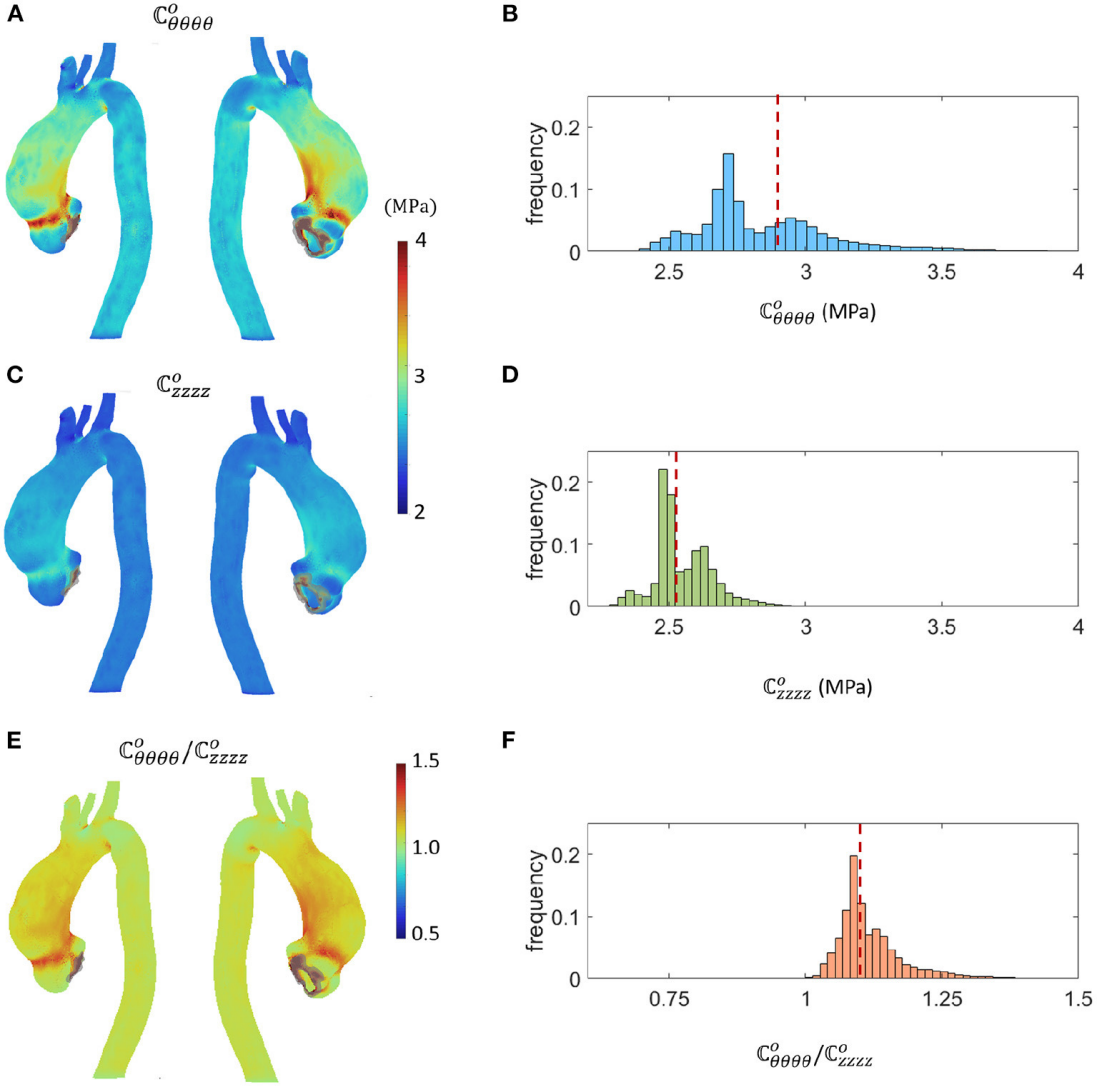
Results from the LD simulation in terms of **(A,B)** ${\mathbb{C}}_{\theta \theta \theta \theta }^{o}$, **(C,D)** ${\mathbb{C}}_{zzzz}^{o}$ and **(E,F)** ${\mathbb{C}}_{\theta \theta \theta \theta }^{o}/{\mathbb{C}}_{zzzz}^{o}$ ratio. Outlier terms were grayed in the maps. Dashed lines in the bar plot distributions highlight the mean values.

The linearized circumferential (${\mathbb{C}}_{\theta \theta \theta \theta }^{o}$), longitudinal (${\mathbb{C}}_{zzzz}^{o}$) stiffness values and their ratio obtained with the SOL-Linearized simulation at the LD step are shown in Figures 5A–E (maps) and Figures 5B–F (barplot). Significant anisotropy and regional variations appear in these results.

**Figure 6:**
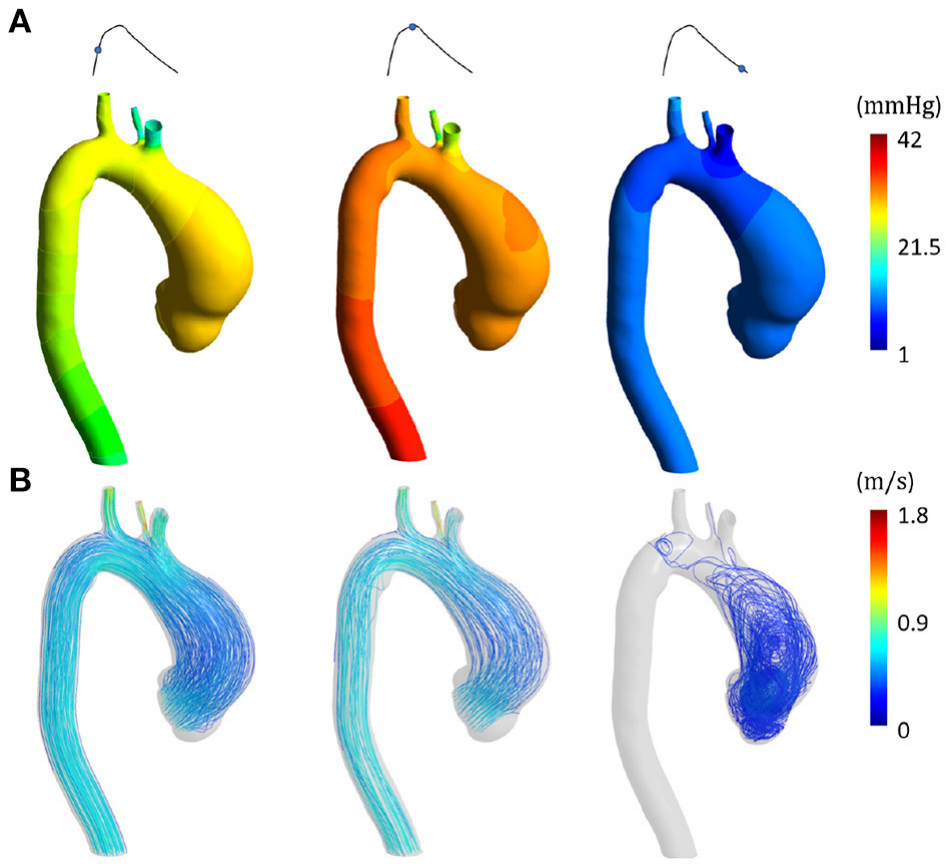
Fluid dynamic results from SOL FSI SD step at early systole, peak systole and late diastole in terms of **(A)** pressure distribution and **(B)** velocity streamlines.

**Figure 7:**
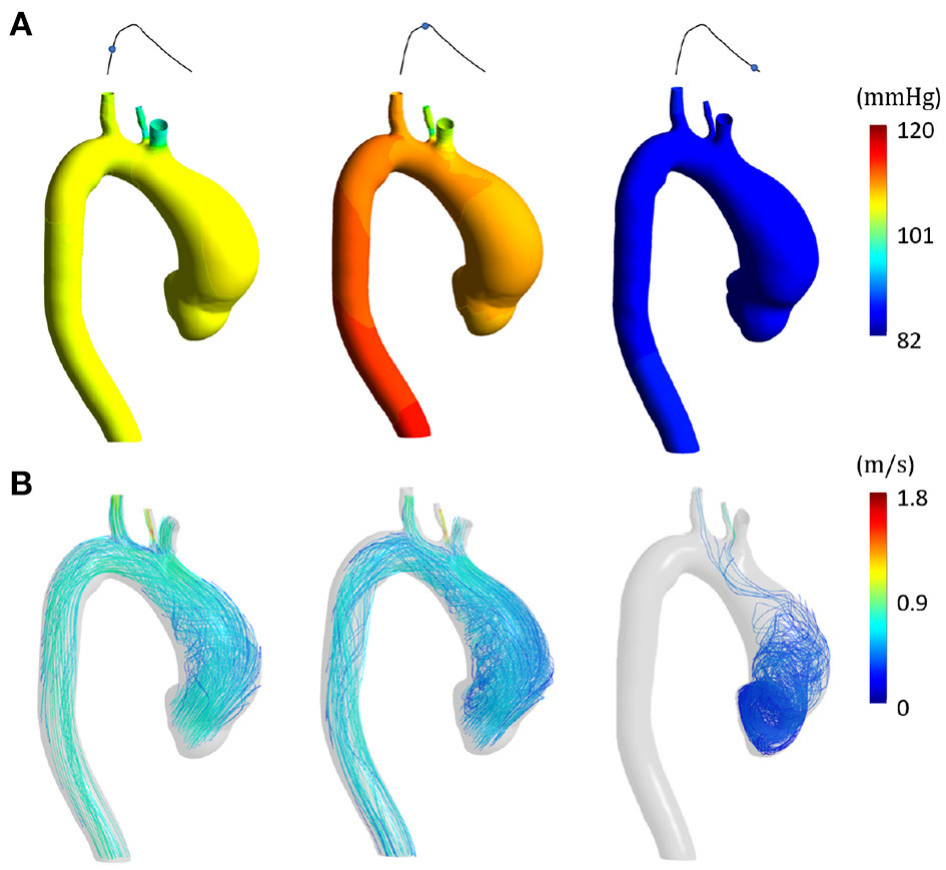
Fluid dynamic results from non-linear FSI at early systole, peak systole and late diastole in terms of **(A)** pressure distribution and **(B)** velocity streamlines.

Figures 6, 7 represent the fluid dynamic conditions obtained from the SOL FSI and the non-linear FSI simulations, respectively. Different cardiac phases are reported: early systole, peak systole and late diastole. As correctly imposed, the pressure ranges are the same except for a pressure shift, representing the diastolic load, which is absent for the SOL case.

**Figure 8:**
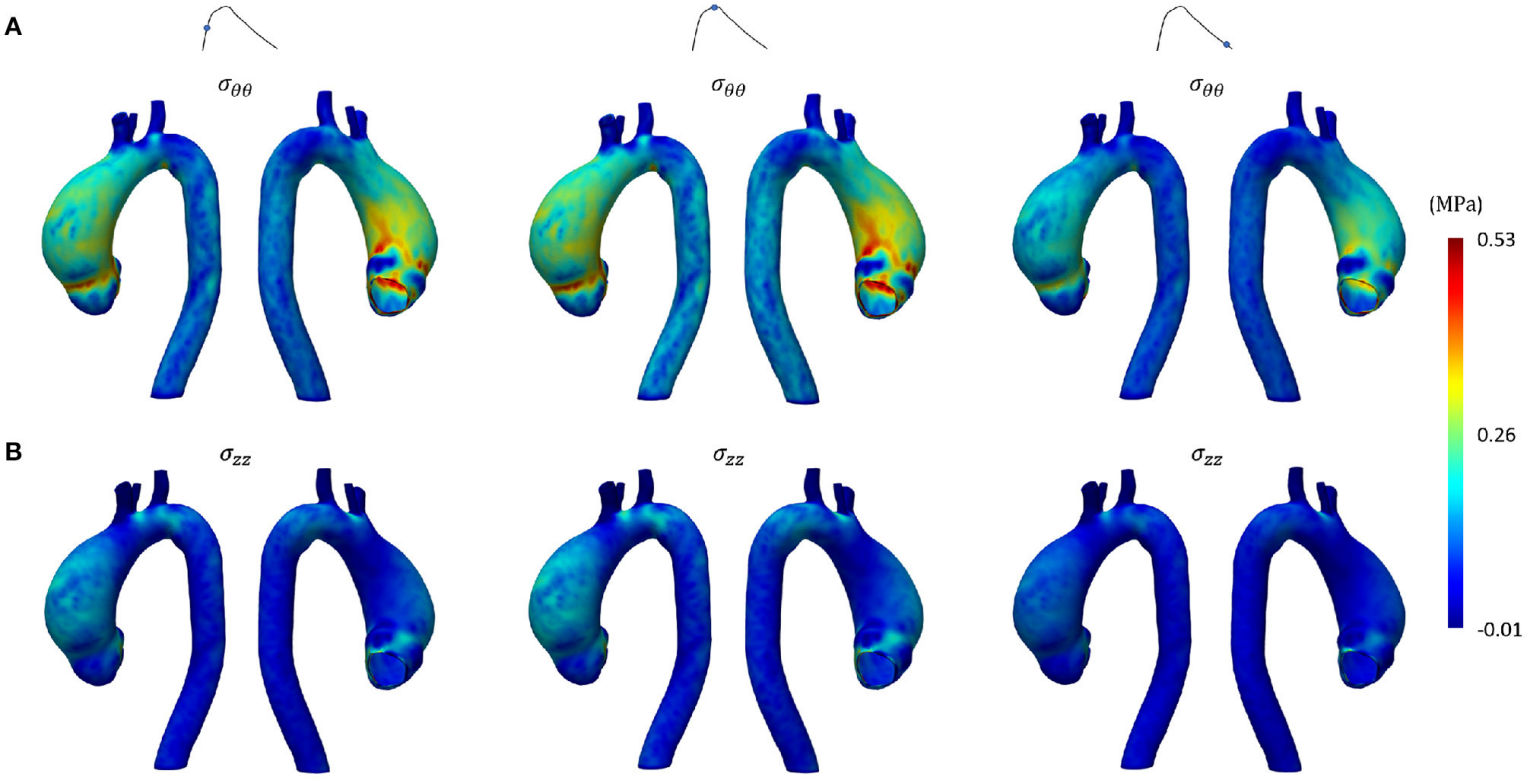
Structural results from SOL FSI SD step at early systole, peak systole and late diastole in terms of **(A)** circumferential and **(B)** longitudinal stress.

**Figure 9:**
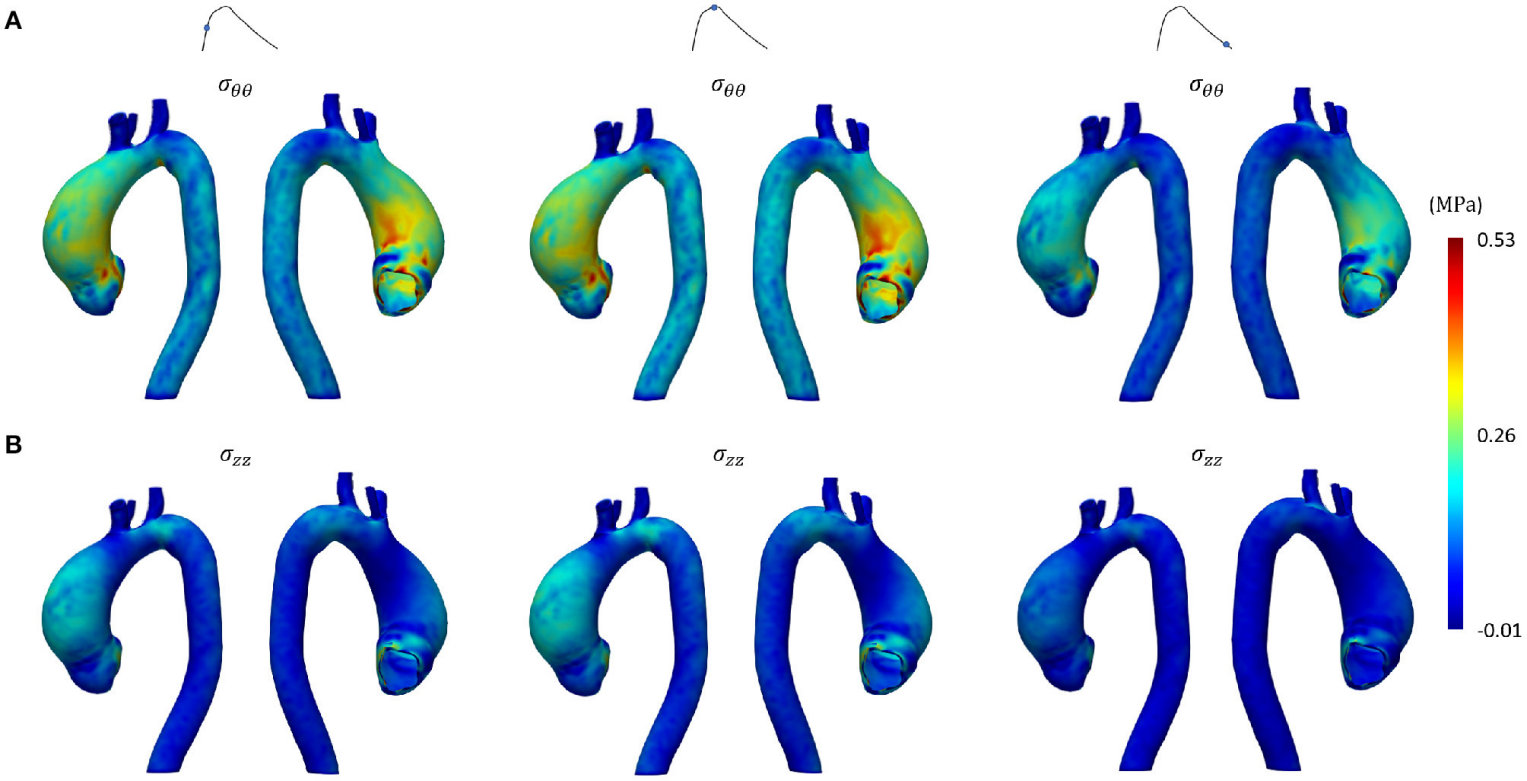
Structural results from non-linear FSI at early systole, peak systole and late diastole in terms of **(A)** circumferential and **(B)** longitudinal stress.

**Figure 10:**
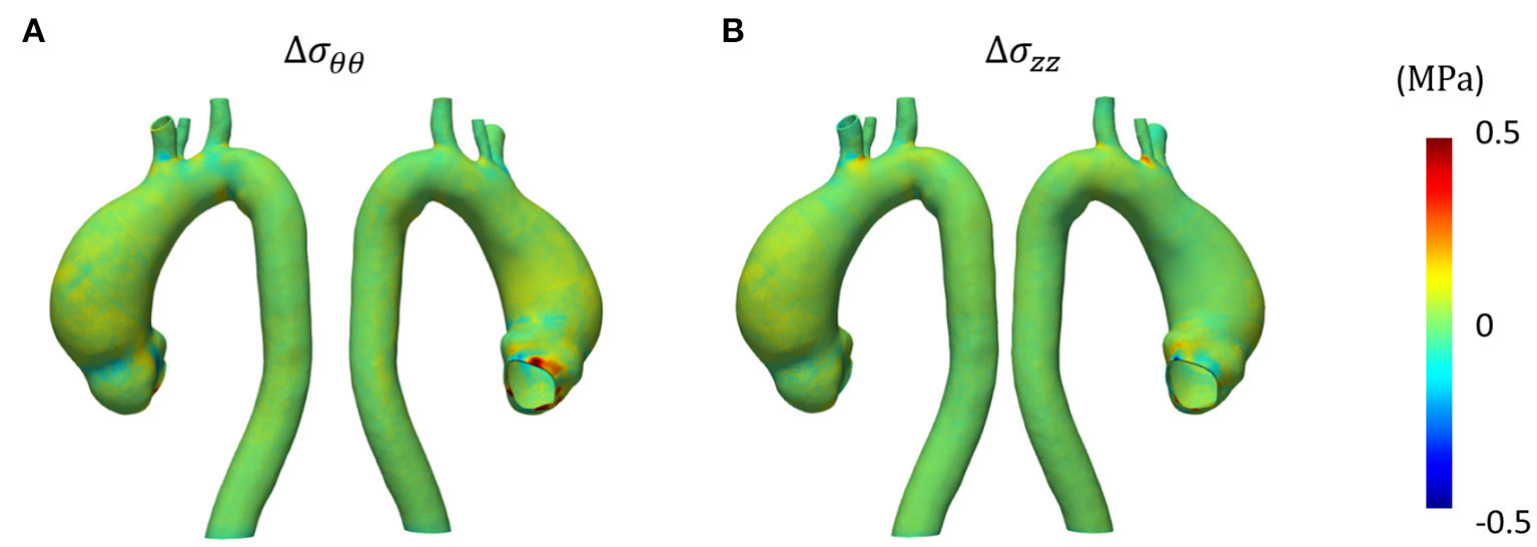
Point-wise difference in terms of circumferential **(A)** and longitudinal **(B)** stress at peak systole.

**Figure 11:**
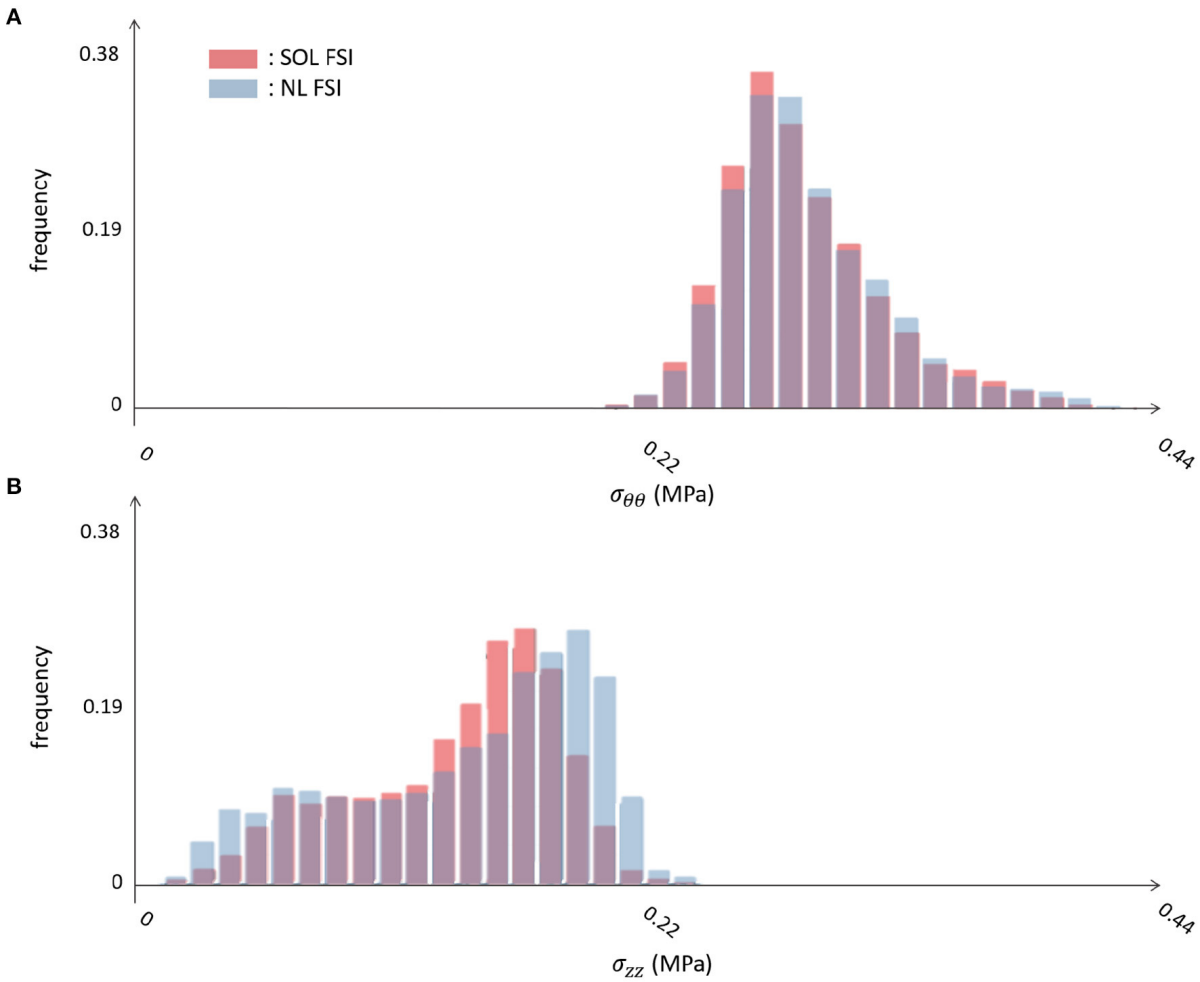
Comparison of distributions, according to the SOL and non-linear FSI simulations, in terms of **(A)** circumferential and **(B)** longitudinal stress in the aTAA region.

Figures 8, 9 represent the structural results from the SOL FSI and the non-linear FSI simulations, respectively. Both circumferential (σ_θθ_) and longitudinal (σ_*zz*_) Cauchy stresses are reported at early systole, peak systole and late diastole. Additionally, the maps reporting the point-wise differences at peak systole both for σ_θθ_ and σ_*zz*_ values are depicted in Figure 10, for the sake of method comparison. Figure 11 represents the σ_θθ_ and σ_*zz*_ distributions in the aTAA region for both the SOL FSI and the non-linear FSI simulations. Table 3 reports the average circumferential ($\bar{\sigma_{\theta \theta }}$) and longitudinal ($\bar{\sigma_{zz}}$) stresses within the aTAA section at peak systole, together with the computational time (*t*_*c*_) for a single cardiac period in regime conditions for both the SOL and the non-linear FSI.

**Table 3:** Table summarizing the average circumferential ($\bar{\sigma_{\theta \theta }}$) and longitudinal ($\bar{\sigma_{zz}}$) stresses at peak systole, with the computational time for a single regime period (*t*_*c*_) for the SOL and non-linear FSI.

	**SOL FSI**	**non-linear FSI**	**Relative error**
$\bar{\sigma_{\theta \theta }}$	0.25 MPa	0.26 MPa	3.8%
$\bar{\sigma_{zz}}$	0.13 MPa	0.14 MPa	7.1%
	**SOL FSI**	**non-linear FSI**	**Time reduction**
*t*_*c*_	6.3 h	12.1 h	47.9 %

## 5. Discussion

The results confirmed that the proposed method is suitable for the SOL linearization in a patient-specific aTAA. The framework was implemented successfully by starting from patient-specific features, including morphology and material properties. The linearized approach of the SOL theory was demonstrated to be feasible for the fully-coupled FSI study of the aTAA case. The results from the analysis were presented with the aim to demonstrate the effectiveness of the SOL approach, allowing the introduction of anisotropic hyperelastic constitutive model in the FSI context with a significant reduction of computational load.

The algorithm for estimating the unloaded condition geometry of the aTAA produced meaningful results. Figure 4 demonstrated the effectiveness of the approach. The initial guess performances (Figure 4A), in which the displacement field is not weighted (*k* = 1), revealed distance errors with peaks of 3.3 mm in the aTAA region. On the other hand, the final result of the algorithm (Figure 4B) showed an improved estimation of the unloaded aTAA morphology, as the distance error in the aTAA region was below 1 mm. The maximum errors were located in the supra-aortic vessel region. These errors can be explained by the imposed boundary conditions and by the fact that the proposed reverse displacement algorithm only accounts for size modifications. Nevertheless, the region of interest of the aTAA is not affected by reconstruction errors. Therefore, the estimation with *k* = 0.802 was chosen as suitable for the FSI analyses of the successive steps.

The linearized stiffness values resulting from the LD simulation phase of the SOL workflow are reported in Figure 5C. As expected, outlier stiffness values were encountered within constrained elements and consequently ignored for the successive SD simulation step. The ${\mathbb{C}}_{\theta \theta \theta \theta }^{o}$ map and bar plot from Figures 5A,B show a heterogeneity within the aTAA section, in agreement with previous studies revealing the local distribution of tissue properties (Bersi et al., 2019; Spronck and Humphrey, 2019). The results demonstrated that the tissue has a stiffer behavior along the θθ direction, with a range from 2.2 to 3.5 MPa. This trend reveals that, even if a homogeneous hyperelastic material model is assumed, significant heterogeneities of tangent stiffness can still arise during the loading of the vessel. The maximum stiffness value was localized at the inner curvature of the aTAA and in the sino-tubular junction, in agreement with previous literature (Campobasso et al., 2018). It is also worth emphasizing that stiffness values were more dispersed along the circumferential direction than along the longitudinal direction. The result is directly derived at the LD step from Equation (13). The ${\mathbb{C}}_{\theta \theta \theta \theta }^{o}/{\mathbb{C}}_{zzzz}^{o}$ distribution in Figures 5E,F reveals that the ratio is larger than 1 in each element, confirming that the tissue is stiffer along the θθ direction. This behavior highlights the anisotropy of the aTAA tissue and the strong link between the aTAA morphology and the current mechanical state of the tissue (Choudhury et al., 2009).

Concerning the SD step and the comparison between the SOL and the non-linear FSI approach, the fluid dynamics results reported in Figures 6, 7 confirmed that our loading conditions were appropriate. The physiological ranges of pressure were correctly reached through the set of lumped parameters, in both cases: 1–42 mmHg for the SOL FSI and, 82–120 mmHg for the non-linear FSI. The structural results from the SOL FSI and the non-linear FSI, reported in Figures 8, 9 respectively, both confirmed the trend from the LD simulation step. As expected, the circumferential stress range remains above the longitudinal stress one. In both simulations, the peak σ_θθ_ stress values are confined within the inner curvature of the aTAA, as highlighted in the stiffness distribution map from the LD step. Concerning the σ_*zz*_ distribution, maximum values are instead reached within the external curvature section. It is worth highlighting that the same stress ranges are reached by both the SOL Linearized FSI and the non-linear FSI, in terms of σ_θθ_ and σ_*zz*_. The point-wise comparison of the stress maps, reported in Figure 10, confirms the trends of Figures 8, 9. The only non-negligible errors are confined around the constrained boundaries. The aTAA section instead exhibits negligible differences both in terms of σ_θθ_ and σ_*zz*_. The stress distributions within the atAA section at the peak systole (Figure 11), allowed a comparison of the SOL method with the non-linear FSI. Kolmogorov-Smirnov non parametric tests confirmed that there were no significant difference between the stress values resulting from SOL and non-linear FSI simulations, with a significance of 5%. The absence of difference was assessed for both σ_θθ_ (*p* = 0.32) and σ_*zz*_ (*p* = 0.11). The comparison between the SOL and non-linear FSI approach was also performed by evaluating average stresses in the aTAA at peak systole, as reported in Table 3. SOL FSI results were in good agreement with the non-linear approach, with only a negligible underestimation. The difference produced inaccuracies of 3.8% and 7.1% for $\bar{\sigma_{\theta \theta }}$ and $\bar{\sigma_{zz}}$, respectively. Nevertheless, the SOL linearization introduced relative errors below 10%, while the computational times were significantly reduced from 12.1 h to 6.3 h for a single regime cycle. On the basis of these data, the approximation introduced by the SOL linearization approach remained marginal in comparison with the gains in terms of computational time reduction.

These results are consistent with other applications of the SOL method in cardiovascular biomechanics (Baek et al., 2007; Ramachandra and Humphrey, 2019). A possible extension of the SOL framework would be to integrate growth and remodeling effects, as done previously by Figueroa et al. (2009). Nevertheless, previously published analyses remained limited to simple vessel morphologies, whereas the current study is the first application to a patient-specific aTAA.

In the current study, some limitations and points of improvement can be highlighted. Currently, the numerical simulations do not account for ventricular motion at the aortic root. The SOL approach was only established on a single patient-specific clinical case for the proof of concept. As future work, it would be interesting to increase the number of analyzed morphologies. Nevertheless, the feasibility of the approach on aTAA was correctly demonstrated. A reference FSI simulation, in which no linearization approximation was introduced, was setup for the sake of comparison. However, a comparison with *in vivo* data would still be valuable. The possibility to validate the flow profiles from *in vivo* 4D flow MRI data would especially be of great interest (Mariotti et al., 2019). Moreover, the adoption of techniques like local extensional stiffness identification or pulse wave velocity approaches could confirm the SOL linearization estimations (Farzaneh et al., 2019; Di Giuseppe et al., 2021). Further validations could be provided for the zero pressure geometry estimation as well. Alternative methods, like the iterative approach from Bäumler et al. (2020) could be used to introduce not only size but also shape modifications for the estimation of the zero-pressure configuration. To this aim, it would also be useful to obtain an aortic pre-stretch measurement during the surgical phase. The stretch measurement could be obtained by introducing optical markers within the ascending aorta before excising the aTAA. This practice would permit to assign more realistic pre-stretch values in the mechanical model. Eventually, we assumed homogeneous hyperelastic properties across the whole aTAA in the current study and no growth and remodeling phenomena were considered. Nevertheless, local stiffness heterogeneity was demonstrated to exist in the aTAA region (Choudhury et al., 2009). We recovered such stiffness heterogeneity through the local variations of stretches but considering also the regional variations of hyperelastic parameters is a potential step further for the SOL approach, these local material properties variations possibly resulting from mechanobiological effects of tissue growth and remodeling (Mousavi et al., 2021).

## 6. Conclusion

In summary, we presented a new method based on the Small On Large linearization to perform patient-specific aTAA fluid-structure simulations at low computational cost, taking into account pre-stress and anisotropy. The comparison with a fully non-linear model was very satisfactory in terms of stress distributions. Future work will focus on validating the method on a large sample of cases, as the computational assets of such methodology can significantly benefit to clinical diagnoses and decisions.

## Data Availability Statement

The original contributions presented in the study are included in the article, further inquiries can be directed to the corresponding author.

## Ethics Statement

The studies involving human participants were reviewed and approved by Fondazione Toscana Gabriele Monasterio, Massa, Italy. The patients/participants provided their written informed consent to participate in this study.

## Author Contributions

SC and SA: conceptualization and supervision. EV: implementation. EV and EG: method and code refinement and validation. SC, EV, and EG: data resources. SC and EV: writing–original draft preparation. EV, EG, SC, and SA: writing–review and editing. All authors contributed to the article and approved the submitted version.

## Conflict of Interest

The authors declare that the research was conducted in the absence of any commercial or financial relationships that could be construed as a potential conflict of interest.

## Publisher's Note

All claims expressed in this article are solely those of the authors and do not necessarily represent those of their affiliated organizations, or those of the publisher, the editors and the reviewers. Any product that may be evaluated in this article, or claim that may be made by its manufacturer, is not guaranteed or endorsed by the publisher.
